# Advanced Wound Care Strategies in Patients with NSTIs

**DOI:** 10.3390/jcm14103514

**Published:** 2025-05-17

**Authors:** Taylor Miller, Jaclyn Clark

**Affiliations:** R Adams Cowley Shock Trauma Center, Baltimore, MD 21201, USA; jaclyn.clark@som.umaryland.edu

**Keywords:** wound care, wound healing, necrotizing soft tissue infections, postoperative care, surgical wound infections

## Abstract

Necrotizing soft tissue infections (NSTIs) are rapidly progressive, life-threatening infections associated with significant morbidity and mortality. Surgical debridement, the cornerstone of treatment, often results in extensive, complex wounds located in anatomically difficult regions. Management of these wounds can be challenging, especially for surgeons with limited experience in complex wound care and reconstruction. Yet, proper management of these wounds is critical to patient recovery and long-term quality of life. This review provides a comprehensive overview of current strategies in NSTI wound reconstruction. It begins by outlining the biological underpinnings of wound healing and the unique challenges posed by NSTI-related wounds. The review then explores a range of dressing materials and advanced wound care modalities, including negative pressure wound therapy, cellular and tissue-based products, and hyperbaric therapy. Finally, it presents a guide to surgical reconstruction techniques, including skin grafting and flap coverage. By consolidating current knowledge and practical guidance, this review seeks to support generalist and acute care surgeons with the knowledge needed to optimize wound healing, enhance functional outcomes, and improve quality of life for NSTI survivors.

## 1. Introduction

Necrotizing soft tissue infections (NSTIs) represent a spectrum of rapidly progressive infections characterized by tissue necrosis, systemic toxicity, and high mortality rates. Despite advances in care, NSTIs remain a significant clinical challenge due to their unpredictable presentation, their rapid progression, and the need for urgent, aggressive surgical debridement. The resulting wounds are often extensive, anatomically complex, and difficult to manage, and pose significant reconstructive challenges.

After patients recover from critical illness, wound closure and coverage become the most important goals for ultimate recovery. However, many general and acute care surgeons, who are frequently on the front lines of NSTI management, may have limited exposure to contemporary strategies in complex wound reconstruction. In addition, as wound care increasingly depends on multidisciplinary teams, a foundational understanding of wound healing principles and advanced wound management techniques is essential for all providers involved in the treatment of these patients.

Given the evolving landscape of wound care technologies and reconstructive options, a timely synthesis of current evidence and best practices is warranted. This review aims to bridge the gap between acute surgical management and definitive wound reconstruction by reviewing the biological principles of wound healing and factors affecting healing in the context of NSTIs, describing commonly used dressing materials and advanced wound care strategies, and outlining surgical reconstruction techniques relevant to NSTI wound closure. By providing a practical, evidence-informed guide, this review seeks to support generalist surgeons and other members of the care team in delivering high-quality, evidence-based wound care for NSTI patients.

## 2. Process of Wound Healing

There are three major stages of wound healing: inflammation, proliferation, and remodeling. The stages overlap and are dependent on one another.

### 2.1. Inflammation Stage

After a wound is created, the inflammation stage is triggered by clot formation and hemostasis and takes place over the first four to six days. Platelets are activated by vascular disruption and release pro-thrombotic cytokines and chemokines which attract neutrophils as part of the immune response. They also release growth factors that attract fibroblasts. Platelets are critical to building the fibrin network that serves as a scaffold for initial wound healing [1]. Multiple signaling mechanisms promote vasodilation, vascular permeability, and edema as part of the inflammatory response. Neutrophils serve as initial innate immune system protection and are soon replaced by macrophages that clean debris and pathogens to prepare the wound for subsequent healing. Macrophage concentration peaks around seven days after injury [2]. Chronic wounds often do not heal because they are unable to move past the inflammatory phase [1].

### 2.2. Proliferative Stage

Characterized by epithelialization, angiogenesis, and formation of granulation tissue, the proliferative stage of wound healing starts around day four and lasts until around day fourteen of wound healing. Epithelial cells proliferate from the wound edges to ultimately cover the wound. Simultaneously, endothelial cells rapidly proliferate in the wound bed to form capillaries and ensure blood supply to the healing tissue. Fibroblasts across the wound bed perform two major functions: production of collagen, which forms granulation tissue, and later differentiation into myofibroblasts to aid in wound bed contraction [3].

### 2.3. Remodeling Stage

The remodeling stage starts around day eight of wound healing and continues for as long as a year. It is characterized by collagen deposition and remodeling. Collagen production is upregulated for four to five weeks during healing. An initial thin collagen layer is deposited parallel to the wound bed for protection, but it is subsequently absorbed and remodeled to reinforce areas of stress on the wound. The wound reaches about 3% of final strength in the first week, 30% after three weeks, and 80% by three months. It will never be as strong as the original skin [1].

When the three stages are complete, wounds are healed and epithelialized.

## 3. Factors Associated with Poor Wound Healing

Unfortunately, NSTIs are associated with numerous risk factors that impair wound healing, all of which must be managed to optimize recovery. Generally, wound size should be measured frequently and wound area should decrease by about 15% per week [4]. A slower rate of closure should prompt re-evaluation of risk factors such as nutritional status, blood supply, or persistent infection.

### 3.1. Infection and Necrotic Tissue

After initial debridement and eradication of infection, there can be a spectrum of secondary bacterial presence in wound beds. Contamination with bacteria refers to the presence of bacteria that are not actively replicating, as can be seen in any open wound and does not affect healing. Bacterial colonization of a wound refers to the presence of replicating bacteria that do not cause infection or immune response. This generally does not interfere with wound healing. At the point of critical colonization, replicating bacteria are numerous enough to delay the healing process. Obtaining a wound biopsy can be helpful in diagnosis of significant colonization and direct treatment, which often includes antibiotic therapy in addition to mechanical debridement. Wound infection refers to the stage at which the patient exhibits an immune response leading to classic clinical signs of infection [4].

Presence of sufficient bacteria in the wound bed halts the wound healing process in the inflammation phase. Vascular permeability and edema are maintained, endotoxins destroy collagen structures, and progress in wound healing is inhibited [1]. Bacteria in the wound bed also take up important nutrients and oxygen that are needed for the wound healing process. Generally, wounds with fewer than 10^5^ bacteria per gram of tissue will progress from the inflammation stage of wound healing to the proliferative phase, but those with more bacteria will have healing halted in the inflammation stage, resulting in a chronic wound [4]. Necrotic tissue becomes a nidus of bacterial growth and prevents wound bed contraction and healing. Multiple international and national society guidelines stress the importance of interdisciplinary care, proper wound dressing, wound reassessment, and exudate management in addressing wound infection [5,6,7,8,9,10]. Despite the effects of infection on wound healing, a short (<7-day) course of antibiotics after source control appears equivalent to longer courses for NSTIs and is given a Category 2B recommendation by the Surgical Infection Society 2020 Guidelines [10].

### 3.2. Perfusion and Ischemia

Wound healing is an energy-intensive process, requiring significant aerobic adenosine triphosphate (ATP) production. Localized hypoxia increases inflammation and vascular permeability, triggers endothelial cell apoptosis, and inhibits the function of neutrophils and fibroblasts [1]. Care must be taken to ensure an adequate blood supply to remaining tissue during debridement. Hyperbaric oxygen is another strategy that can be used to promote perfusion and healing.

### 3.3. Edema

Localized tissue edema increases tissue pressures and can prevent normal perfusion. This contributes to localized ischemia. NSTI patients often receive large volumes of fluids as part of their resuscitation and are at especially high risk of both localized and generalized edema. Additionally, acute or chronic lymphatic drainage impairment can affect a tissue’s ability to effectively drain. External compression and wrapping can aid this process [1].

### 3.4. Diabetes

The effect of diabetes on wound healing is multifactorial. Diabetes itself contributes to vascular insufficiency. Hyperglycemia additionally delays wound healing through a myriad of mechanisms. In particular, nonenzymatic glycosylation of collagen has been shown to significantly affect collagen remodeling as well as fibroblast function [1]. Both the Centers for Disease Control and Prevention (CDC) and World Health Organization Guidelines give a strong recommendation for intensive blood glucose control (blood glucose levels < 200 mg/dL) in both diabetic and nondiabetic patients to minimize surgical site infection [9,11].

### 3.5. Age

Wound healing is slower in older patients [1]. The causes of this are multifactorial, but related to other risk factors associated with age such as vascular disease, poor perfusion, and poor immune response. 

### 3.6. Smoking

Smoking is a major modifiable risk factor for poor wound healing. The mechanism is largely related to reduced perfusion to the wound bed, and studies have shown a lower partial pressure of oxygen in tissue related to smoking [1]. In addition to the vasoconstrictive effects of nicotine, smoking also has been shown to reduce proliferation of cells related to wound healing and deposition of collagen [1].

### 3.7. Sepsis and Multiorgan Failure

Sepsis refers to a dysregulated host immune response to infection. This has obvious implications for the inflammatory response involved in wound healing. Sepsis also results in poor oxygen delivery and tissue perfusion, dysregulated coagulation, and increased vascular permeability and localized tissue edema. All of these factors can interfere with the normal wound healing process.

### 3.8. Nutrition

Adequate nutrition is vital to wound healing. NSTI patients are often critically ill and at high risk for malnutrition because of both poor nutritional intake as well as a hypermetabolic and catabolic state associated with their critical illness. Wound healing is thought to require at least 30–35 kcal/kg per day, and patients with large wounds are thought to require 250% more protein and 50% more calories than other patients [12]. The amino acids arginine and glutamine, vitamins A, C, and E, and the minerals zinc, selenium, and iron are thought to be essential to wound healing [12].

## 4. Wound Management

### 4.1. Surgical Debridement

**Figure 1:**
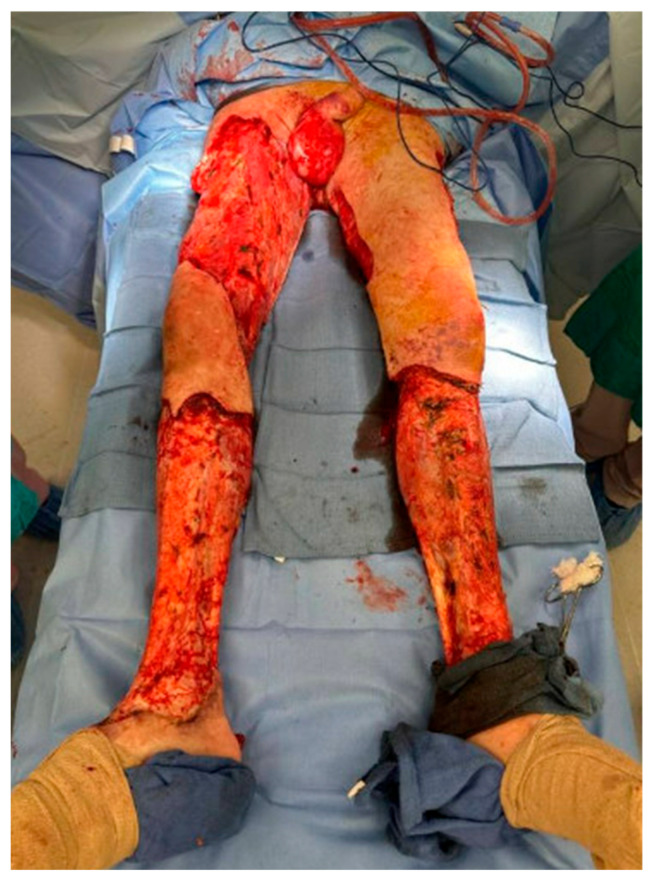
Large wounds created by massive debridement for NSTI.

Prompt surgical debridement of necrotic tissue is the backbone of NSTI care, as has been established in multiple national and international guidelines [13,14,15]. This might require multiple visits to the operating room, ensuring not only that obviously necrotic tissue is removed, but that devitalized ischemic tissue has also been debrided. After fulfilling these criteria, a healthy-appearing clean wound bed will become the best substrate for wound healing (Figure 1). The strategies and devices used in caring for these often large wounds act to augment the biologic process of wound healing. Thus, proper care of NSTI surgical wounds starts with technique in the operating room.

### 4.2. Wound Dressings

The ultimate purpose of wound management is for wounds to be closed and epithelialized to allow patients to recover from their NSTIs. There are unfortunately very few high-quality data to guide physicians in choosing the optimal wound dressing for a specific wound. Many data that exist are extrapolated from studies of chronic wounds and diabetic ulcers. The Global Guidelines for the Prevention of Surgical Site infection by the World Health Organization, based on a systematic review of the literature, reported no difference in outcomes based on surgical dressing used, with a low quality of evidence [16].

Because of the lack of evidence to guide dressing choice, wound dressings are generally chosen to address the specific needs of each individual wound. When choosing a dressing, physicians should consider the size and depth of a wound, the amount of exudate, the characteristics of the wound bed (granulation, stage of healing, presence of necrotic tissue), risk of infection, and anatomic location. The general goals addressed by wound dressings are listed below [4,16,17].

Purposes of Wound Dressings:

- Protecting wounds from mechanical damage;
- Protecting wounds from infection;
- Addressing dead space;
- Providing a moist wound bed;
- Absorbing excess exudate;
- Minimizing trauma from dressing changes;
- Allowing frequent re-evaluation of the wound;
- Protection of surrounding skin.

Thus, dressing choice is driven by wound appearance, edema, patient ability to tolerate dressing changes, resource utilization requirements, and patient values [16].

Moist Gauze Dressing: “Wet-to-Dry”

Ensuring a moist wound bed is considered the standard of care for healing of open wounds [5]. The moist environment promotes epithelialization, angiogenesis, and collagen deposition. Moisture protects protein-rich wound exudate, which provides nutrients, allows diffusion of growth factors and migration of cells, and participates in autolysis of dead tissue. Because of this, moist wounds heal 2–3 times faster than dry wounds [4,18]. Packing a wound with moist gauze covered by a dry protective layer is a common technique that is accessible and inexpensive. The packing eliminates dead space. As the wet gauze dries, it adheres to the underlying surface, which is removed when the dressing is changed. This is beneficial in that it allows residual devitalized tissue and bacteria-colonized biofilm to be removed but can be harmful if granulation tissue is also removed. Because gauze can adhere to underlying tissue, moist wound dressings should not be placed directly in contact with fragile structures without an underlying protective nonadherent dressing [4]. Moisture can be provided by simple saline, but antiseptic solutions such as sodium hypochlorite (Dakin solution), hypochlorous acid (Vashe), and sodium oxychloroscene (Clorpactin) can all be used to control bacterial contamination in wounds. Dressing changes can be performed frequently, or as necessary to provide gentle debridement of the wound. While these dressing changes can be relatively inexpensive, they can be painful and time-consuming depending on the surface area to be covered.

Ideal for packing of large wounds, dressing between interval wound debridements, and management of highly exudative wounds;

Avoid in the following cases: avoid allowing gauze to dry and adhere to fragile structures.

Impregnated (Low-Adherent) Gauze

Low-adherent gauze dressings are impregnated with specific materials such as petroleum or zinc to maintain moisture and protect underlying tissue. They are nonadherent, which protects the underlying tissue from damage from other wound dressings. Some of these dressings have antimicrobial components as well [4,16].

Brand names and common types: Xeroform, Adaptic, and paraffin dressing;

Ideal for protection of fragile tissues; can be used under moist gauze;

Avoid in excessively exudative wounds.

Films

Transparent films are polyurethane semi-occlusive dressings. This property allows some gas exchange (water vapor and oxygen) but retains water, exudate, and autolytic proteins. They are transparent, which facilitates wound re-evaluation. Because they are semi-permeable, they are not able to efficiently handle excessive moisture [4,16,17].

Brand names: Opsite, Tegaderm, and Biooclusive;

Ideal for epithelializing wounds;

Avoid in excessively exudative wounds.

Hydrogels

Hydrogels are crosslinked hydrophilic polymers that can absorb up to 96% water. They promote a moist environment and can support autolytic debridement. They are best used to hydrate a dry wound or eschar but can also absorb some amount of wound exudate. However, they can lead to wound bed maceration if used on heavily exudative wounds. Data on hydrogel dressings are sparse, with systematic reviews finding differing results. One Cochrane Review did find evidence that hydrogels are superior to normal dressings in healing diabetic foot ulcers, but another found no evidence of improved healing in pressure ulcers [19,20]. Hydrogels are also available in silver-impregnated forms [4,16,17,19].

Brand names: Intrasite, Nugel, Aquaform, ActiformCool, and Aquaflo;

Ideal for dry wounds and granulating wounds;

Avoid in excessively exudative wounds.

Hydrocolloids

Hydrocolloid dressings consist of colloids such as gelatin, pectin, or carboxymethylcellulose which can be combined with a polyurethane film dressing. When the colloid comes in contact with wound exudate, it forms a gel that is nonadherent and impermeable to water and water vapor. Because of this property, these dressings effectively maintain moisture and protect the wound bed. They support autolytic debridement, granulation, and epithelialization [16,17].

Brand Names: Granuflex, NUDERM, and Aquacel;

Ideal for granulating and epithelializing wounds, and dry eschars;

Avoid in excessively exudative wounds and infected wounds.

Foams

Foam dressings are made primarily of hydrophilic polyurethane foam that absorbs exudate while maintaining a moist environment. They are often combined with an adhesive, occlusive outer layer. Foam is primarily used for its absorptive properties in highly exudative wounds, but can dry out wounds with little or no exudate [17,21].

Brand Names: Silastic, Mepilex, Lyofoam, Tegaderm, Hyperfoam, Hydrocell, Allevyn, and Biatain;

Ideal for exudative wounds;

Avoid in dry wounds and infected wounds.

Alginates and Hydrofibers

Alginates are made from seaweed and are highly absorbable. Their ability to absorb 15–20 times their weight in fluid makes them especially useful for heavily exudative wounds [17]. Alginates turn into a biodegradable gel when combined with moisture, which can be helpful for filling dead space in large wounds [4,21]. The addition of silver to alginate dressing has been shown to improve bacterial status on biopsies of wounds but not evidence of infection [22].

Hydrofibers are made from silver carboxymethylcellulose and serve a similar function to alginates. They are highly absorbent and form a gel when in contact with a wound bed. The common brand name Aquacel combines a hydrofiber dressing with silver for antimicrobial properties [17,23,24].

Alginate brand names: Sorbsan, Kaltostat, CarraSorb, AlgiDERO, Algisite, Curasorb, and SeaSorb;

Hydrofiber brand names: Aquacel;

Ideal for heavily exudative wounds;

Avoid in dry wounds.

Antimicrobial Dressings

Common antimicrobial dressings include silver-, iodine-, or honey-containing dressings [21]. Silver has very effective antibacterial properties while not being toxic to human cells. It is effective against bacteria that are resistant to antibiotic medications such as MRSA and VRE, and no known resistance has developed [4]. Silver dressings also save resources by reducing the frequency of required dressing changes. A silver-impregnated sponge can be used with NPWT, or a black sponge can be lined with silver-impregnated dressings to take advantage of silver’s antibacterial effects [4]. Silver-releasing foam dressings have been shown to accelerate wound healing rates [25,26]. Although two older Cochrane Reviews in 2010 did not find sufficient evidence to conclude that silver-containing dressings were beneficial, multiple more recent meta-analyses have found benefits in both rates of wound healing and infectious complications [16,22,25,26,27,28,29,30]. Honey is similarly effective against many pathogens, including MRSA, VRE, and fungi. The mechanism of its antimicrobial properties is thought to be related to high osmolarity, facilitation of H_2_O^2^ production, and viscosity, which provides a protective barrier [23].

Negative Pressure Wound Therapy

**Figure 2:**
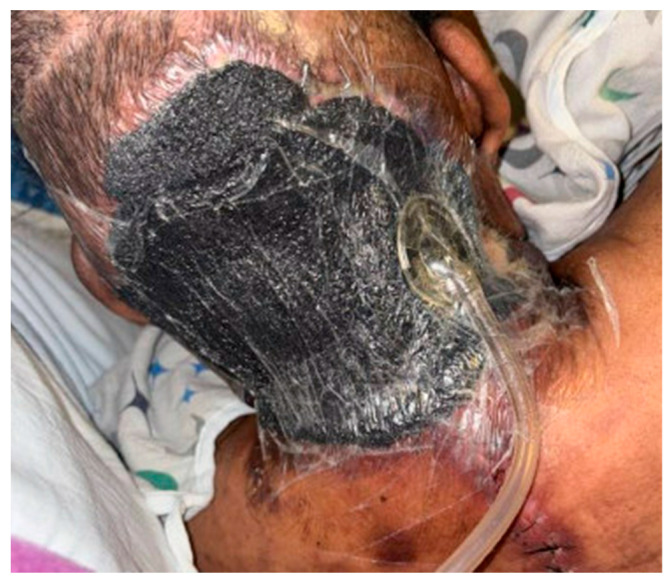
NPWT used on the back of the scalp and neck.

Negative pressure wound therapy (NPWT) uses a regulated vacuum device to deliver subatmospheric pressures (usually 125 mmHg) across a wound bed (Figure 2). On a macroscopic level, NPWT allows contraction of large wounds with elimination of dead space and application of uniform pressure across the wound bed [31]. NPWT additionally allows management of excess fluid, removal of exudative fluids and proteins, bacterial clearance, and promotion of angiogenesis, circulation to the wound bed, and granulation. The NPWT device is thought to create microdeformations and strain at the tissue level that promote fibroblast proliferation as well as increased production of VEGF and IL-8, promoting angiogenesis [31]. One study demonstrated that application of NPWT improved blood flow to the affected tissues, increased the rate of granulation tissue formation, and reduced bacterial counts after four days [32]. A study looking at negative pressure therapy in NSTI wounds specifically found lower mortality rates (10.7% vs. 16.2%), and lower rates of open wounds at discharge (52.7% vs. 81.5%) with patients using traditional NPWT compared to moist dressings, though there was a high risk of selection bias in this retrospective review [33]. NPWT has also been shown to be helpful in patients with surgical wound infections, both in increasing the proportion of patients with healed wounds and in decreasing hospital length of stay [26]. It can be very helpful in the management of wounds with excessive exudate [18]. Additional advantages include ease of application and spacing out of dressing changes to every three days. The Surgical Infection Society 2020 guidelines give a Category 2C recommendation to use NPWT after adequate debridement to facilitate wound healing [10].

A major disadvantage of NPWT, especially in lower-resource settings, is cost. Multiple cost-effectiveness studies have shown, however, that NPWT can be more cost-effective than other methods of wound therapy due to faster wound healing, better outcomes, and reduced need for dressing changes. For example, one study of long-term acute care patients with complex wounds found that those treated with NPWT had more rapid wound area reduction per day (1.08 cm^2^ per day vs. 0.19 cm^2^ per day) compared to those treated with moist dressings despite having larger initial wounds. Despite having a higher total cost, the cost per cubic centimeter reduction in wound volume was significantly lower in the NPWT group (USD 11.90/cm^3^ vs. USD 30.92/cm^3^) [34].

### 4.3. Other Advanced Wound Care Options

Hyperbaric Therapy

There are conflicting data on the effects of hyperbaric oxygen therapy on mortality in patients with NSTIs, with some observational data suggesting possible benefit but no quality randomized trials [35,36,37,38,39]. In addition to its purported mortality benefit, hyperbaric therapy may promote wound healing for NSTI patients. Multiple pathways by which hyperbaric therapy could expedite wound healing have been posited, including increased angiogenesis and ability to deliver oxygen past capillaries blocked by NSTI-related microthrombi [4]. Though small, one randomized trial found both increased survival and increased rates of limb salvage with the use of hyperbaric therapy in NSTI patients [36]. There are significant randomized data that hyperbaric therapy increases rates of non-NSTI-related wound healing, is effective for diabetic ulcers, and can rescue threatened flaps [37]. In 2017, the Tenth European Consensus Conference on Hyperbaric Medicine gave a Type 1 recommendation of using hyperbaric therapy for NSTI patients based on Level C evidence [38]. However, a Cochrane Review and the Surgical Infection Society both find insufficient evidence to recommend hyperbaric therapy [10,40].

Nanoparticles

The use of nanoparticles holds significant promise in the management of infected surgical wounds. Nanoparticles have several unique properties due to their size. They have a larger surface area-to-volume ratio, have increased bioavailability, and are often small enough to penetrate bacterial cell walls. This allows them to have antimicrobial effects while limiting cytotoxic effects to human cells.

There are multiple different types of nanoparticles, each with different properties. Metal nanoparticles, especially silver and gold, have direct antimicrobial effects and have the most robust (though still limited) evidence behind their use. Their antimicrobial effects are exerted through multiple, poorly understood, pathways, including inhibition of DNA replication, interference with bacterial proteins, and damage to cell membranes. Silver nanoparticles, especially, do little damage to human cells. Randomized trials of silver nanoparticles show faster wound healing, lower bacterial counts, and less biofilm formation with the use of these particles [41].

Other nanoparticle types include glass, carbon-based, lipid-based, and polymer nanoparticles. They induce their antimicrobial effects through various mechanisms, from direct action on pathogens to serving as a vehicle for enhanced drug delivery. Further research is needed to better understand optimal use of these nanoparticles.

## 5. Reconstructive Surgical Techniques

**Figure 3:**
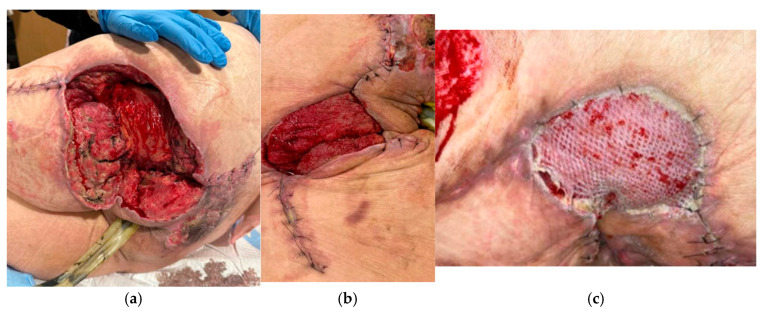
(**a**) Left hip wound after partial primary closure and rotational muscle flap. (**b**) Same wound after NPWT. (**c**) Ultimate skin grafting.

The reconstructive ladder describes surgical techniques used to promote wound closure in a hierarchy from least to most sophisticated. Generally, the simplest appropriate reconstructive technique should be chosen. The steps on the ladder are listed below, and results can be seen in Figure 3 [42,43].

Primary Closure

Clean surgical wound edges that can be approximated without tension can undergo primary closure. This usually involves deep absorbable sutures with a layer of superficial nonabsorbable sutures. The wound is then covered in a clean dressing. This is not an appropriate option if there is concern about persistent wound infection, which can worsen if the wound is closed. Oftentimes, a wound might undergo partial closure of as much as will close without tension, creating the smallest open wound possible. This can be performed in a delayed fashion as well [43].

Healing by Secondary Intention

Healing by secondary intention involves a wound being left open without an attempt at primary closure. Over time, the wound becomes smaller and contracts. This is a slow process and often does not achieve full epitheliazation, especially in the case of large wounds. This approach is successful when the wounds are dressed and kept clean, and is used when a wound cannot be primarily closed due to tension, lack of tissue, or infection. Modifiable risk factors for poor wound healing should be addressed. All wound care and dressing strategies can be applicable for this type of wound [43].

Skin Substitutes

There are several products that can be placed prior to skin grafting to augment skin grafting success. They can be biologically derived acellular or cellular, from humans or animals like cows, pigs, or fish, or synthetic, and function as a dermal and/or epidermal substitute. There are many of these products available. Integra^®^, the first skin substitute created in the 1980s, still is commonly used today for covering avascular aspects of wounds, such as tendon or bone, and allows subsequent skin grafting. Kerecis, an acellular freeze-dried codfish skin-based acellular product used to accelerate healing, has shown promise for faster wound healing [44]. Another such product is NovoSorb™ biodegradable temporizing matrix (BTM). This is a synthetic dermal scaffold that has been bioengineered to mimic the dermis and epidermis. It can be used on both vascular and avascular tissue, has lower cost relative to other such products, and has demonstrated good outcomes in the NSTI population [45]. All of these products act as scaffolds for deposition of necessary proteins and glycoproteins necessary for ultimate wound healing. In addition to cost, a downside to these products is the need to monitor for adherence to underlying tissue, which can take several days with NPWT and involve multiple trips back to the operating room.

Skin Grafts

*Split thickness skin grafting* is an option to promote skin coverage for large wounds that cannot be primarily closed, are too big for healing by secondary intention, and do not involve dead space. These grafts contain epithelium and some dermal cells. Donor sites are usually the thighs, legs, or torso and can heal without requiring closure. These skin grafts require fixation under a dressing for approximately five days to allow integration of the graft [42,43]. While they cover large areas and provide epitheliazation, the cosmetic result is variable.

*Full thickness skin grafting* is an option for smaller wounds, especially in cosmetically important areas. These skin grafts include the epithelium and full thickness dermis. They experience less contraction and often have a better cosmetic outcome. The donor site is closed via primary closure. These are not a good option in very large wound beds, as obtaining the graft creates another open wound that needs to be closed.

Flaps

Flap reconstruction is the coverage of a wound with autologous tissue that keeps its original vascular supply. Flaps can contain a variety of combinations of muscle, subcutaneous fat, and skin. Rotational flaps elevate tissue but do not disconnect the main blood supply, and relocate the tissue to a nearby wound bed. These are often helpful in neck and chest, abdomen, and pelvic/hip wounds. Free flaps are also a possibility, though not performed as often for open wounds. These involve severing the main blood supply of the tissue to be transposed, and relocation to a distant site of the body [42]. In general, flap reconstruction requires a specialist, often a plastic surgeon, to perform. Wounds need to be clean in order to have the best chance for flap survival.

## 6. Future Directions

As this review highlights, surgical debridement is the beginning of a long, complex healing process for NSTI patients. This review attempts to bridge the gap between surgical management of this disease and long-term wound management for the acute care surgeon and other members of the interdisciplinary care team. Understanding of the pathophysiology of wound healing, risk factors for poor wound healing, and advanced wound care techniques is important for managing these patients. Future directions in wound care are likely to include smart wound dressings that can give the care team real-time information about the wound bed and further innovation in skin substitutes [46,47,48]. Just as important as scientific innovations, however, will be innovations in pain management and rehabilitation systems that support patients in achieving high-functioning recovery.
